# Efficacy of 1.5% Dish Washing Solution and 95% Lemon Water in Substituting Perilous Xylene as a Deparaffinizing Agent for Routine H and E Staining Procedure: A Short Study

**DOI:** 10.1155/2014/707310

**Published:** 2014-03-31

**Authors:** Anuradha Ananthaneni, Srilekha Namala, Vijay Srinivas Guduru, V. V. S. Ramprasad, Sabitha Devi Ramisetty, Urmila Udayashankar, Kiran Kumar Naik

**Affiliations:** Department of Oral and Maxillofacial Pathology, St Joseph Dental College, Duggirala, Eluru, Andhra Pradesh 534004, India

## Abstract

*Aim*. To assess the efficacy of dish washing solution and diluted lemon water in deparaffinizing sections during conventional hematoxylin and eosin staining technique. *Objective*. The objective is to utilize eco-friendly economical substitute for xylene. *Materials and Methods*. Using twenty paraffin embedded tissue blocks, three sections each were prepared. One section was stained with conventional H and E method (Group A) and the other two sections with xylene-free (XF) H and E (Groups B and C). Staining characteristics were compared with xylene and scoring was given. Total score of 3–5 was regarded as adequate for diagnosis and less than that inadequate for diagnosis. *Statistical Analysis*. Chi-square test, Kruskal Wallis ANOVA test, and Mann-Whitney *U* test were used. *Results*. Adequacy of nuclear staining, crispness, and staining for diagnosis were greater in both Groups A and C (100%) than Group B (95%). Adequacy of cytoplasmic staining was similar in all the three groups (100%). Group B showed comparatively superior uniform staining and less retention of wax. *Conclusion*. Dish washing solution or diluted lemon water can be replaced for xylene as deparaffinizing agent in hematoxylin and eosin procedure.

## 1. Introduction

Xylene, also known as xylol or dimethylbenzene, is an aromatic synthetic hydrocarbon that forms an imperative part of pathological laboratory since many years. It is available naturally in coal tar and petroleum and has derived its name from crude wood spirit (Greek xylon- wood). It is colorless, flammable liquid or gas with a sweet smell. Three forms of xylene exist called isomers in which the methyl groups vary on the benzene ring:* meta-*xylene,* ortho-*xylene, and* para-*xylene (m-, o-, and p-xylene). Laboratory-grade xylene is composed of m-xylene (40–65%), p-xylene (20%), o-xylene (20%), and ethyl benzene (6–20%) and traces of toluene, thiophene, trimethyl benzene, phenol, hydrogen sulfide, and pyridine. It is used as a clearing agent in tissue processing where it causes maximum displacement of alcohol and makes the tissue transparent thus enhancing paraffin infiltration and as a deparaffinizing agent in staining and cover slipping. Although it is extremely useful, it leads to health hazards when exposed involving almost all parts of the body ranging from nausea, vomiting to death (Table 1). Current Occupational Safety and Health Administration permissible exposure limit for xylene is 100 ppm as an 8-hour time-weighted average (TWA) concentration. Limonene reagents, aliphatic hydrocarbon mixtures, aromatic hydrocarbon mixtures, and mineral oil mixtures were used as alternatives for xylene in tissue processing as clearing agent. But peak exposure is during dewaxing of sections. Unrelenting usage of xylene is a paradigm of its futile replacement [1–5].

It enters the body by means of lungs and is stored in adipose tissue (due to its solubility in it) especially in the subcutaneous fat with a half life of 1 to 6 days, with long-term exposure leading to permanent disability caused by diminution of mitochondrial adenosine triphosphate in the affected cells [4]. In liver, it is metabolized by oxidation of a methyl group and conjugation with glycine to yield methyl hippuric acid that is excreted in the urine. It is intricate to dispose. It can seep out into the surface water, soil, or ground water where it may remain for months or more before it breaks down into other chemicals [1]. Besides occupational exposure, the prime pathway of human contact is through soil contamination. Hence the present study is intended to replace xylene with nonbiohazardous agents like dish washing solution (DWS) and diluted lemon water (DLW).

## 2. Aim

The aim of the present study is to assess the efficacy of DWS and DLW as a deparaffinizing agent for conventional hematoxylin and eosin (CHE) procedure by comparing their staining characteristics with those of xylene.

## 3. Objective

To reduce the exposure of xylene during dewaxing, less biohazardous and economical agents like DWS and DLW were substituted for xylene in the present study.

## 4. Materials and Methods

Twenty paraffin embedded tissue blocks from our department were obtained. Three sections of 4 microns thick were prepared from each block. One section was stained with CHE method where xylene was used as deparaffinizing agent. The other two sections were stained with XF hematoxylin and eosin (H and E), where 1.5% DWS (1.5 mL dish washing solution in 98.5 mL distilled water) and 95% DLW (95 mL lemon water in 5 mL of distilled water) were used as deparaffinizing agent.

*Group A.* Tissue sections which were stainedwith CHE method.

*Group B*. Tissue sections which were stainedwith XF H and E where 1.5% DWS was used as deparaffinizing agent.

*Group C*. Tissue sections which were stainedwith XF H and E where 95% DLW was used as deparaffinizing agent.

Each section was scored and evaluated by a single oral pathologist who was blinded. Slides were checked for parameters, nuclear staining, cytoplasmicstaining (adequate = score1, inadequate = score0), uniformity, clarity, and crispness (present = score1, absent = score0). Total score less than or equal to two was inadequatefor diagnosis and 3–5 was adequate for diagnosis. The presence or absence of wax in stained sections was also recorded. Chi-square test, Kruskal Wallis ANOVA test, and Mann-Whitney *U* test were the tests used for statistical analysis.

## 5. Results

It was noted that standard time period of the H and E staining procedure ranged from 50 to 55 minutes for Group A (Table 2), 25 to 30 min for Group B (Table 3), and 54 min for Group C (Table 4).

**Figure 1:**
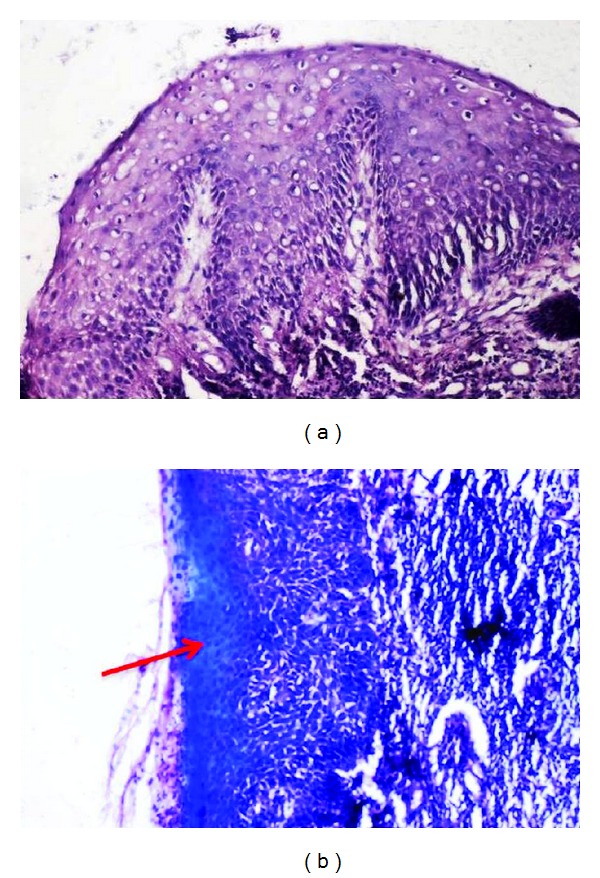
Photomicrograph of Group A stained section (a) and red arrow showing the residual wax (b).

**Figure 2:**
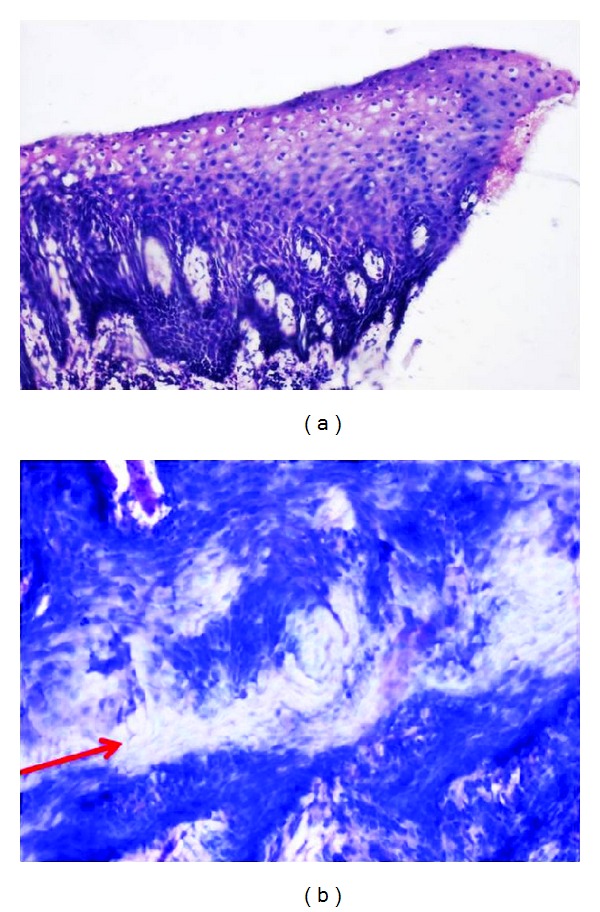
Photomicrograph of Group B stained section (a) and red arrow showing the residual wax (b).

**Figure 3:**
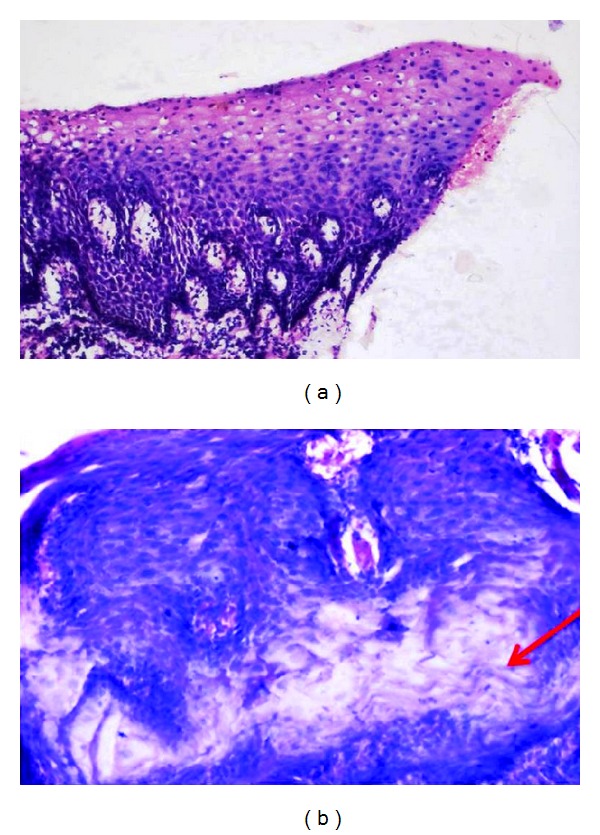
Photomicrograph of Group C stained section (a) and red arrow showing the residual wax (b).

Adequacy of nuclear staining, crispness, and staining for diagnosis were 100% in all the sections in both Groups A and C and 95% in Group B (*P* > 0.05) (Tables 5–7); adequate cytoplasmic staining and clarity were seen in all the sections in all the three groups (*P* > 0.05) (Tables 8 and 9); uniform staining was present in 65% of Group A, 75% of Group B, and 55% of Group C (*P* > 0.05) (Table 10); wax retention was seen in 50% in Groups A and C and 40% in Group B (*P* > 0.05) (Table 11) (Figures 1, 2, and 3).

## 6. Discussion 

Liquid DWS is highly foaming mixture of surfactants principally made up of alkylbenzene sulfonates with low skin irritation and is principally used for hand washing of cutlery, glasses, cooking utensils, and plates [6, 7]. In earlier studies, it was successfully demonstrated as an alternate for xylene in deparaffinizing tissue sections [3, 5, 8]. Lemon juice is customarily used to brighten up copper cookware, as a sanitary kitchen deodorizer, and to remove grease, polish, and wood cleaner, and so forth. Review of literature showed no study till date where DLW was used as deparaffinizing agent. The novel concept of using DLW as deparaffinizing agent was from its solvent property used to dissolve old wax [9].

The underlying principle is that the high temperature of 90 to 94°C will help in removing the wax and dish wash by its surfactant property reduces the surface tension whereas lemon water by its solvent property prevents the wax from resticking onto the slides, thus helping in deparaffinizing the sections.

In the study done by Ankle and Joshi, [5] Mayer's hematoxylin was used which is a progressive stain whereas in our study and study done by Ramulu et al. [3] Harri's hematoxylin was used which is a regressive stain. Ramulu et al. [3] demonstrated that Harri's hematoxylin can also be used instead of Mayer's hematoxylin.

The study showed that out of 20 sections, adequate nuclear staining was noted in all the sections in both Group A and 95% in Group B (Table 5). Adequate cytoplasmic staining and clarity were noted in all the sections in all the three groups (*P* > 0.05) (Tables 8 and 9); uniform staining was present in 75% of Group B and 65% of Group A (*P* > 0.05) (Table 10) but the difference was not significant indicating that there is no difference between the two staining methods. This is in contrast with Ankle and Joshi [5] and Ramulu et al. [3] where Group B showed comparatively superior adequacy in nuclear staining, cytoplasmic staining, and clarity and inferior uniformity than Group A.

Crisp staining and adequacy of staining for diagnosis were seen in 95% in Group B and 100% in Group A (*P* > 0.05) (Tables 6 and 7) which are similar to results seen in Ramulu et al. [3] whereas, in the experiment done by Ankle and Joshi [5], Group B showed upgradation in crisp staining and adequacy for diagnosis compared to Group A.

Retention of wax is higher in both Groups A and C (50%) when compared to Group B (40%) (*P* > 0.05) (Table 11) (Figures 1–3).

100% of sections of Group C showed adequate nuclear staining, cytoplasmic staining, clarity, crisp staining, and adequacy of stained sections for diagnosis whereas 55% showed uniform staining (*P* > 0.05) (Tables 5–11).

Time taken for dewaxing with DWS and DLW was comparatively less with 4 and 21 min, respectively, whereas with xylene it took 25 min.

Both DWS and DLW have more advantages when compared to xylene like being nontoxic, nonbiohazardous, easy to dispose, and less costly. Even though DLW has an added advantage of natural availability, time taken by it to dewax the sections was more (21 min) when compared to DWS (4 min) (Table 12).

The disadvantages of XF H and E were that the procedure is temperature sensitive and requires electricity. It cannot be done if power supply is not available. Slight drop in temperature leads to improper removal of wax from sections, and on the other hand increase in temperature would lead to lifting up and loss of sections from slides. As it is known that clearing agent has to be miscible with both alcohol and wax but since the XF method employs hydrophilic agents it cannot be used as a clearing agent for substituting xylene.

Besides, these few elite disadvantages can be used for xylene more readily than sheltering behind a dangerous agent.

## 7. Conclusion

The current study results evidenced that 1.5% dish washing solution and 95% diluted lemon water can admittedly substitute xylene as deparaffinizing agents in H and E procedure.

## Figures and Tables

**Table 1:** Health hazards of xylene (Kandyala et al. [1]).

SNO	System	Effect
1	Nervous system	100–200 ppm → nausea and headache.
		200–500 ppm → dizziness, weakness, and vomiting.
		800–10,000 ppm → giddiness, confusion, slurred speech, loss of balance, and ringing sound.
		>10,000 ppm → sleepiness, loss of consciousness, and death.

2	GIT	Nausea, vomiting, and gastric discomfort.

3	ENT	Irritation and damage to eye (accidental splash).

4	Muscle	Reduced grasping power and reduced muscle power in extremities.

5	Skin	Irritation, dermatitis, dryness, and flaking and cracking of skin.

6	Cancer	Carcinogenic in animals.

7	Reproductive	Delayed ossification and contaminates breast milk.

8	Lungs	Irritation, chest pain, and shortness of breath (200 ppm).
		Pulmonary edema (extreme conditions).

9	Liver and kidney	Injury.

**Table 2:** Routine H and E staining procedure.

Deparaffinization and Rehydration	Xylene	25 min
	90% alcohol	1 min
	70% alcohol	1 min
	Water wash	1 min

Nuclear staining	Harris hematoxylin	8 min
	Tap water wash	2 min

Differentiation	Differentiation in 1% acid alcohol	1 dip

Bluing	1% lithium carbonate	1 min
	Water wash	10 min

Cytoplasmic staining	1% eosin	1 min

Dehydration	70% alcohol	30 sec
	90% alcohol	30 sec
	Xylene	1 min
	Approximate time required	50 to 55 min

**Table 3:** Xylene and alcohol free H and E staining procedure where diluted dish wash was used as deparaffinizing agent.

Deparaffinization	Diluted dish washing soap 1.5%-I	At 90°C	1 min
	Diluted dish washing soap 1.5%-II	At 90°C	1 min
	Distilled water-I	At 90°C	30 sec
	Distilled water-II	At 90°C	30 sec
	Wash slides in distilled water	At 45°C	30 sec
	Wash slides in distilled water	At room temperature	30 sec

Nuclear staining	Harris hematoxylin	At room temperature	8 min
	Tap water wash		2 min

Differentiation	Differentiation in 1% acid alcohol	At room temperature	1 dip

Bluing	Tap water wash		10 min

Cytoplasmic staining	1% eosin	At room temperature	1 min
	Tap water wash		1 min
	Wash slides in distilled water		

Dehydration	Over drying the sections	At 60°C	10 min
	Approximate time required		25 to 30 min

**Table 4:** Xylene and alcohol free H and E staining procedure using 95% lemon water as deparaffinizing agent.

Deparaffinization	Diluted lemon water 95%-I	At 94°C	5 min
	Diluted lemon water 95%-II	At 94°C	5 min
	Distilled water-I	At 94°C	5 min
	Distilled water-II	At 94°C	5 min
	Wash slides in distilled water	At 45°C	30 sec
	Wash slides in distilled water	At room temperature	30 sec

Neutralizing the effect of acidity in lemon water	Lithium carbonate		15 min
	Tap water wash		5 min

Nuclear staining	Harris hematoxylin	At room temperature	2 min
	Tap water wash		2 min

Differentiation	Differentiation in 1% acid alcohol	At room temperature	1 dip

Bluing	Tap water wash		10 min

Cytoplasmic staining	1% eosin	At room temperature	1 dip
	Tap water wash		1 min
	Wash slides in distilled water		

Dehydration	Over drying the sections	At 60°C	5 min
	Approximate time required		54 min

**Table 5:** Adequacy of nuclear staining.

Nuclear staining	Group A	%	Group B	%	Group C	%	Total	%
Adequate	20	100.0	19	95.0	20	100.0	59	98.3
Inadequate	0	0.0	1	5.0	0	0.0	1	1.7
Total	**20**	**100.0**	**20**	**100.0**	**20**	**100.0**	**60**	**100.0**

Chi-square = 2.0342, df = 2, *P* = 0.3617								
Among all groups, Kruskal Wallis ANOVA, *H* = 2.0000, *P* = 0.3170								
Between Group A and Group B, Mann-Whitney *U* test, *Z* = −1.0000, *P* = 0.0780								
Between Group A and Group C, Mann-Whitney *U* test, *Z* = 0.0000, *P* = 1.0000								
Between Group B and Group C, Mann-Whitney *U* test, *Z* = 1.0000, *P* = 0.3170								

**Table 6:** Adequacy of crispness of staining.

Intensity of staining	Group A	%	Group B	%	Group C	%	Total	%
Present	20	100.0	19	95.0	20	100.0	59	98.3
Absent	0	0.0	1	5.0	0	0.0	1	1.7
Total	**20**	**100.0**	**20**	**100.0**	**20**	**100.0**	**60**	**100.0**

Chi-square = 2.0342, df = 2, *P* = 0.3617								
Among all groups, Kruskal Wallis ANOVA, *H* = 2.0000, *P* = 0.3170								
Between Group A and Group B, Mann-Whitney *U* test, *Z* = −1.0000, *P* = 0.0780								
Between Group A and Group C, Mann-Whitney *U* test, *Z* = 0.0000, *P* = 1.0000								
Between Group B and Group C, Mann-Whitney *U* test, *Z* = 1.0000, *P* = 0.3170								

**Table 7:** Adequacy of stained sections for diagnosis.

Score	Group A	%	Group B	%	Group C	%	Total	%
Adequate	20	100.0	19	95.0	20	100.0	59	98.3
Inadequate	0	0.0	1	5.0	0	0.0	1	1.7
Total	**20**	**100.0**	**20**	**100.0**	**20**	**100.0**	**60**	**100.0**

Chi-square = 2.0342, df = 2, *P* = 0.3617								
Among all groups, Kruskal Wallis ANOVA, *H* = 2.0000, *P* = 0.3170								
Between Group A and Group B, Mann-Whitney *U* test, *Z* = −1.0000, *P* = 0.0780								
Between Group A and Group C, Mann-Whitney *U* test, *Z* = 0.0000, *P* = 1.0000								
Between Group B and Group C, Mann-Whitney *U* test, *Z* = 1.0000, *P* = 0.3170								

**Table 8:** Adequacy of cytoplasmic staining.

Cytoplasmic staining	Group A	%	Group B	%	Group C	%	Total	%
Adequate	20	100.0	20	100.0	20	100.0	60	100.0
Inadequate	0	0.00	0	0.00	0	0.00	0	0.00
Total	**20**	**100.0**	**20**	**100.0**	**20**	**100.0**	**60**	**100.0**

Chi-square = 0.0000, df = 2, *P* = 1.0000								
Among all groups, Kruskal Wallis ANOVA, *H* = 0.0000, *P* = 1.0000								
Between Group A and Group B, Mann-Whitney *U* test, *Z* = 0.0000, *P* = 1.0000								
Between Group A and Group C, Mann-Whitney *U* test, *Z* = 0.0000, *P* = 1.0000								
Between Group B and Group C, Mann-Whitney *U* test, *Z* = 0.0000, *P* = 1.0000								

**Table 9:** Adequacy of clarity of staining.

Clarity of staining	Group A	%	Group B	%	Group C	%	Total	%
Present	20	100.0	20	100.0	20	100.0	60	100.0
Absent	0	0.00	0	0.00	0	0.00	0	0.00
Total	**20**	**100.0**	**20**	**100.0**	**20**	**100.0**	**60**	**100.0**

Chi-square = 0.0000, df = 2, *P* = 1.0000								
Among all groups, Kruskal Wallis ANOVA, *H* = 0.0000, *P* = 1.0000								
Between Group A and Group B, Mann-Whitney *U* test, *Z* = 0.0000, *P* = 1.0000								
Between Group A and Group C, Mann-Whitney *U* test, *Z* = 0.0000, *P* = 1.0000								
Between Group B and Group C, Mann-Whitney *U* test, *Z* = 0.0000, *P* = 1.0000								

**Table 10:** Adequacy of uniformity of staining.

Uniformity of staining	Group A	%	Group B	%	Group C	%	Total	%
Present	13	65.0	15	75.0	11	55.0	39	65.0
Absent	7	35.0	5	25.0	9	45.0	21	35.0
Total	**20**	**100.0**	**20**	**100.0**	**20**	**100.0**	**60**	**100.0**

Chi-square = 1.7582, df = 2, *P* = 0.4151								
Among all groups, Kruskal Wallis ANOVA, *H* = 1.7290, *P* = 0.4210								
Between Group A and Group B, Mann-Whitney *U* test, *Z* = 0.6810, *P* = 0.4960								
Between Group A and Group C, Mann-Whitney *U* test, *Z* = −0.6370, *P* = 0.5240								
Between Group B and Group C, Mann-Whitney *U* test, *Z* = −1.3090, *P* = 0.1900								

**Table 11:** Retention of wax.

Intensity of staining	Group A	%	Group B	%	Group C	%	Total	%
Not retained	10	50.0	12	60.0	10	50.0	32	53.3
Retained	10	50.0	8	40.0	10	50.0	28	46.7
Total	**20**	**100.0**	**20**	**100.0**	**20**	**100.0**	**60**	**100.0**

Chi-square = 0.5362, df = 2, *P* = 0.76502								
Among all groups, Kruskal Wallis ANOVA, *H* = 0.5270, *P* = 0.7680								
Between Group A and Group B, Mann-Whitney *U* test, *Z* = −0.6280, *P* = 0.5300								
Between Group A and Group C, Mann-Whitney *U* test, *Z* = 0.0000, *P* = 1.0000								
Between Group B and Group C, Mann-Whitney *U* test, *Z* = −0.6280, *P* = 0.5300								

**Table 12:** Advantages of XF H and E method.

	Routine H and E	1.5% DWS	95% DLW
Cost	High	Low	Low
Time	50–55 min	25–30 min	54 min
Toxicity	Present	Absent	Absent
Biohazardous	Yes	No	No
Inflammability of chemicals used	Present	Absent	Absent
Staining protocol	Lengthy	Simplified	Simplified
Handling	Toxic if not properly handled	Easy	Easy
Quality of staining	Good	Good	Good
Disposal of chemicals	Difficult	Easy	Easy
Preparation	Synthetic	Synthetic	Naturally available from plants

## References

[1] Kandyala R, Raghavendra SP, Rajasekharan ST, Xylene: (2010). An overview of its health hazards and preventive measures. *Journal of Oral and Maxillofacial Pathology*.

[2] OSHA (Occupational safety and health administration) Air contaminants occupational safety and health administration. http://www.atsdr.cdc.gov/toxprofiles/tp71-c1.pdf.

[3] Ramulu S, Koneru A, Ravikumar S, Sharma P, Ramesh DNSV, Patel R (2012). Liquid dish washing soap: an excellent substitute for xylene and alcohol in hematoxylin and eosin staining procedure. *Journal of Forensic Sciences*.

[4] Buesa RJ, Peshkov MV (2009). Histology without xylene. *Annals of Diagnostic Pathology*.

[5] Ankle MR, Joshi PS (2011). A study to evaluate the efficacy of xylene-free hematoxylin and eosin staining procedure as compared to the conventional hematoxylin and eosin staining: An Experimental Study. *Journal of Oral and Maxillofacial Pathology*.

[6] http://www.en.wikipedia.org/wiki/Detergent.

[7] http://en.wikipedia.org/wiki/Dishwashing_liquid.

[8] Falkeholm L, Grant CA, Magnusson A, Möller E (2001). Xylene-free method for histological preparation: a multicentre evaluation. *Laboratory Investigation*.

[9] http://en.wikipedia.org/wiki/Lemon.

